# Amino Terminal Acetylation of HOXB13 Regulates the DNA Damage Response in Prostate Cancer

**DOI:** 10.3390/cancers16091622

**Published:** 2024-04-23

**Authors:** Duy T. Nguyen, Urvashi Mahajan, Duminduni Hewa Angappulige, Aashna Doshi, Nupam P. Mahajan, Kiran Mahajan

**Affiliations:** 1 Division of Urologic Surgery, Department of Surgery, Washington University in St. Louis, St. Louis, MO 63110, USA; 2 Mayo Clinic Graduate School of Biomedical Sciences, Jacksonville, FL 32224, USA; 3 A.T. Still University of Health Sciences, Kirksville, MO 63501, USA; 4 Department of Biochemistry, University of Washington, Seattle, WA 98195, USA; 5 Siteman Cancer Center, Washington University in St. Louis, St. Louis, MO 63110, USA

**Keywords:** HOXB13, DNA damage, prostate cancer, acetylation, radio resistance, CBP/p300, condensate

## Abstract

**Simple Summary:**

A significant number of prostate cancers (PC) recur in patients despite treatment with chemotherapy or radiotherapy. While HOXB13 contributes to resistance to anti-androgen treatment, its role in radio resistance is unknown. Herein, we show that HOXB13 assembles at DNA damage sites and colocalizes with γH2AX at double strand breaks despite Androgen Receptor antagonism. Functionally, ablation of *HOXB13* sensitizes PC cells to either radiotherapy or anti-androgen Enzalutamide as well as combination therapies. Resistance to these agents is mediated by acetylation of HOXB13 at lysine 13, which acts as an interaction module for the Switch deficient Sucrose Nonfermenting (SWI/SNF) chromatin remodeling complex. K13-acetylated HOXB13 is required for effective DNA replication following DNA damage and for the formation of nuclear puncta. Our results reveal a hitherto unknown but critical role for HOXB13 in ensuring genomic integrity in PC.

**Abstract:**

Advanced localized prostate cancers (PC) recur despite chemotherapy, radiotherapy and/or androgen deprivation therapy. We recently reported HOXB13 lysine (K)13 acetylation as a gain-of-function modification that regulates interaction with the SWI/SNF chromatin remodeling complex and is critical for anti-androgen resistance. However, whether acetylated HOXB13 promotes PC cell survival following treatment with genotoxic agents is not known. Herein, we show that K13-acetylated HOXB13 is induced rapidly in PC cells in response to DNA damage induced by irradiation (IR). It colocalizes with the histone variant γH2AX at sites of double strand breaks (DSBs). Treatment of PCs with the Androgen Receptor (AR) antagonist Enzalutamide (ENZ) did not suppress DNA-damage-induced HOXB13 acetylation. In contrast, HOXB13 depletion or loss of acetylation overcame resistance of PC cells to ENZ and synergized with IR. *HOXB13K13A* mutants show diminished replication fork progression, impaired G2/M arrest with significant cell death following DNA damage. Mechanistically, we found that amino terminus regulates HOXB13 nuclear puncta formation that is essential for proper DNA damage response. Therefore, targeting HOXB13 acetylation with CBP/p300 inhibitors in combination with DNA damaging therapy may be an effective strategy to overcome anti-androgen resistance of PCs.

## 1. Introduction

Prostate cancer (PC) is the most commonly diagnosed and a leading cause of cancer-related deaths among American men [1]. Surgery, chemotherapy, radiation and androgen deprivation therapy (ADT) are treatment options for men with localized advanced disease [2]. DNA damage-induced tumor ablation by radiotherapy with either external beam radiotherapy, brachytherapy for localized high-risk PC or Lu^177^-PSMA-targeted radioligand therapy has gained traction for recurrent hormone-sensitive and metastatic castration-resistant PCs (CRPCs). Likewise, chemotherapy with DNA-damaging agents, Etoposide or DNA cross-linking agents such as cisplatin or carboplatin are an option for hormone-refractory patients [3,4,5]. However, CRPCs are significantly radioresistant compared to hormone-sensitive PC. Despite the initial response, recurrence is not uncommon in patients treated with the above therapies, suggesting that the benefit of treatment is short-lived. Moreover, from a physician’s perspective, it is unclear which tumors are likely to be radioresistant to recommend patients for surgical radical prostatectomy or prostate-saving radiotherapy [6,7]. Although the pathogenic role of epigenetic regulators and lineage-specific transcription factors (TFs) in driving therapeutic resistance of PC is emerging, their contribution to radio resistance is understudied [8,9,10].

Homeobox B13 (HOXB13), a homeodomain-containing transcription factor, regulates prostate gland differentiation during development and interacts with the Androgen Receptor (AR) to fine-tune its activity at various genomic sites in the normal prostate [11,12]. Deregulated HOXB13 protein expression is observed in ~85% of prostate adenocarcinomas and correlates with biochemical recurrence, metastatic progression after radical prostatectomy and resistance to AR-targeted therapies [13,14,15]. Moreover, increased HOXB13 mRNA expression is associated with lower inflammation and T-cell activation in prostate biopsies of benign prostatic hyperplasia patients [16]. Molecularly, the Bromodomain and Extra-Terminal (BET) protein BRD4 drives *HOXB13* expression in CRPCs through its recruitment to the BRAH 1/2/3 enhancers [10]. Conversely, genetic ablation of *HOXB13* significantly impacts the growth of human prostate xenograft tumors in castrated male mice [10]. Recently, we uncovered that gain-of-function acetylation at lysine (K) 13 at the amino (N)-terminus is essential for direct interaction with the bromodomain protein BRD9, a member of the Switch deficient Sucrose Nonfermenting (SWI/SNF) chromatin remodeling complex [17]. Functionally, this single-site mutation at HOXB13 increases sensitivity to ENZ and inhibits CRPC xenograft tumor growth [17]. Mechanistically, acetylated HOXB13 regulates super enhancer activity at several genes critical for PC growth, including AR, angiopoietin, vascular endothelial growth factor A and ACK1 tyrosine kinase [17]. While these studies underscore a critical role for HOXB13 in supporting transcription and epigenetic regulation in PC [18], whether acetylation regulates DNA damage response to various genotoxic stresses is not known.

SWI/SNF multi-subunit protein chromatin remodeling complex utilizes the energy of ATP hydrolysis to alter chromatin dynamics during nuclear processes [19,20]. While mutations in SWI/SNF members are reported in ~20% of human malignancies, more recent studies indicate a context-dependent role for its members in regulating transcription [19,21,22]. Recently, we uncovered that proteins enriched in the acK13-HOXB13 peptide affinity pulldown included the SWI/SNF proteins (SNF2L1/SMARCC1), the bromodomain-containing proteins (BAZ2B, SMARCA2, BRD7 and BRD9), as well the chromatin looping protein CTCF, with BRD9 as the direct reader of HOXB13 acetylation [17,19]. In contrast, unmodified HOXB13 peptide interacted with the transcription pre-initiation complex, including TAF1A/B/C/D and TAF3 associated with RNA Polymerase II [23,24].

Herein, we report a role for HOXB13 in conferring resistance to DNA double-strand break (DSB)-inducing agents such as irradiation and etoposide. We also tested mitomycin C, a double-stranded DNA alkylating agent as a representative example of agents that covalently crosslink DNA and thereby inhibit DNA strand separation during replication. Some of the stalled replication forks can lead to the formation of one-ended double-strand breaks [25,26]. HOXB13 K13 acetylation levels are increased in response to DNA damage by radiation. Loss of K13 acetylation results in impaired replication and repair of DSBs to sensitize cells to genotoxic stress. We uncovered an unknown nuclear localization signal within the amino terminus of HOXB13 encompassing the lysine 13 acetylation that facilitates HOXB13 nuclear retention and aggregation independent of the DNA binding homeodomain. Expression of the amino-terminal domain is protective against DNA damage-induced cell death. Together, our results reveal an important role for HOXB13 in the maintenance of genome integrity of prostate cancers.

## 2. Materials and Methods

### 2.1. Cell Lines and Cell Culture

VCaP, 22Rv1 and HEK293 were obtained from American Type Culture Collection (ATCC). C4-2B was cultured as described previously [27]. The *HOXB13K13A* site-directed mutant C4-2B and 22Rv1 clones have been reported earlier [17]. C-terminal green fluorescent protein (GFP) tagged HOXB13 N-terminal domain (NTD), HOXB13 DNA binding domain (DBD), HOXB13-full length WT and HOXB13-K13A mutant expressing constructs were generated by Genscript (Piscataway, NJ, USA) for this study. All cultures were tested for mycoplasma contamination every 2 months using the PCR Mycoplasma Test Kit I/C (PromoKine, Heidelberg, Germany). Identities of all cell lines used in the current study were confirmed by Short Tandem Repeat (STR) Profiling.

### 2.2. Chemicals

The following chemicals or inhibitors were purchased from Selleck Chemicals (Houston, TX, USA); A-485 (Cat# S8740)**,** Enzalutamide (Cat# S1250) and Olaparib (Cat# S1060). Etoposide (Cat# E1383), Mitomycin C (Cat# M4287), 5-Chloro-2′-deoxyuridine (Cat# C6891, 5-Iodo-2′-deoxyuridine (Cat# I7125), Dimethyl Sulfoxide (Cat# D8418) and 4′,6-Diamidino-2-phenylindole dihydrochloride (Cat# D9542) were purchased from Sigma-Aldrich (St. Louis, MO, USA). BRD9 inhibitor (Cat# T6859), SWI/SNF inhibitor, AU-15330 (T39954) and bromodomain inhibitor GNE-049 were purchased from TargetMol (Wellesley Hills, MA, USA).

### 2.3. Antibodies

pan-HOXB13 (F-9) (Cat# sc-28333) and pan-p53 antibodies (Clone# DO-1; sc-126) were purchased from Santacruz Biotech (Santa Cruz, CA, USA). β-Actin (AC-74) was purchased from Millipore Sigma (Burlington, MA, USA). HRP-conjugated anti-mouse (Cat# W4021) and HRP-conjugated anti-rabbit (Cat# W4011) were purchased from Promega (Madison, WI, USA). Acetylated HOXB13 antibody has been described earlier [17]. γH2AX phospho-serine 139 (Clone 2F3; Cat# 613402) was purchased from Biolegend (San Diego, CA, USA). p53 serine 15 (Cat# 9284S) and HRP-conjugated anti-rabbit secondary antibodies (Cat# 7074) were purchased from Cell Signaling Technology (Boston, MA, USA). BD™ Purified Mouse Anti-BrdU Cat# 347580) (for IdU) was purchased from BD Biosciences (San Jose, CA, USA). Rat anti-BrdU (Cat# ab6326) was purchased from Abcam (Cambridge, UK).

### 2.4. DNA Damage Analysis

Cells were irradiated with an X-ray machine (Precision X-ray, Bradford, CT, USA) or treated with Etoposide with or without the addition of other inhibitors and harvested at various time points for downstream applications such as immunoblotting, immunofluorescence microscopy, flow cytometry and cell cycle analysis.

### 2.5. Cell Transfection

HEK293T cells were transfected with the vector or HOXB13-GFP-tagged constructs using the X-tremeGENE transfection reagent (Millipore Sigma, Darmstadt, Germany). GFP Expression was confirmed by fluorescence microscopy. Transfected cells were processed for experiments as described in the study.

### 2.6. Immunoblotting Analysis

Frozen cell pellets were lysed by sonication in receptor lysis buffer (RLB) and processed as described [27]. Lysates were quantitated by Bradford protein assay (Bio-Rad, Hercules, CA, USA). Protein extracts (40–60 μg) were size-fractionated on 4–12% Bis-Tris gels or 16% Tris-Glycine gels (histones) and transferred onto PVDF membranes (Immobilon, Darmstadt, Germany). Signals were visualized using a chemiluminescent system, and images were captured using the iBright CL1500 imaging system (Thermo Fisher Scientific, Waltham, MA, USA). All blots shown represent the results of at least two independent experiments.

### 2.7. Immunofluorescence Analysis

A total of 100,000 cells were seeded on six-well plates containing a coverslip at the bottom of each well. Following treatment, cells were fixed with 4% paraformaldehyde, permeabilized in 90% ice-cold methanol or 0.1% Triton X-100 for five minutes at room temperature, washed twice with 1X phosphate-buffered saline (PBS) prior to blocking for 60 min in 5% fetal bovine serum (FBS) and 2% BSA. Cells were then stained in a blocking solution containing primary Ab (anti-γH2AX) for 60 min. After two washes with 1X PBS, cells were stained with Alexa 488 green or Rhodamine-red tagged secondary antibodies for 30 min. Cells were mounted with 4′,6-diamidino-2-phenylindole (DAPI). Images were captured using an EVOS M5000 microscope (Thermo Fisher Scientific, Waltham, MA, USA) and analyzed with ImageJ Version 1.54 or Adobe Photoshop cc 2019.

### 2.8. Clonogenic Survival Assay

Following irradiation at different doses (0–10 Gy), parental or *HOXB13K13A* mutant cell lines were plated in six-well plates in 6 replicates for single cells and cultured in regular media for ~12 days. Cells were fixed with 0.5% crystal violet solution in 25% methanol in 1X PBS for 15 min and washed to rinse off excess dye. Macroscopically visible colonies were counted, and the surviving fraction was calculated as described in [28].

### 2.9. Cell Proliferation Assay

Cells were seeded in 96-well plates containing 200 µL of growth medium per well. In experiments testing sensitivity to Enzalutamide or A-485, Olaparib or GNE-049, eight replicates were included for each concentration. Four days after the addition of the drug, 25 µL of cell titer-Glo 2.0 (Promega) was added to each well, and luminescence was recorded after 15 min using a Synergy HTX multi-mode reader (BioTek, Winooski, VT, USA). The half-maximal inhibitory concentrations (IC_50_) were calculated using GraphPad Prism version 9.0.

### 2.10. DNA Fibre Assay

Cells were plated on day one. Media was aspirated from flasks and washed once with 1X PBS. For analog incorporation on day two, cells were incubated with 5-Iodo-2′-deoxyuridine (IdU) and incubated for 20 min at 37 °C, followed by washing in 1X PBS. Cells were incubated with 5-Chloro-2′-deoxyuridine (CldU) (100 mM-stock in Dimethyl Sulfoxide) and treated for 20 min at 37 °C. Cells were washed once with cold 1X PBS gently followed by trypsinization and neutralization with media. Cells were counted using a hemocytometer and resuspended in cold 1X PBS. For spreading the DNA fibers, two μL of cell suspension (1250 cells/μL) was added to the top of the slide. This was followed by adding six μL of lysis buffer and lysing the cells by pipetting up and down. Samples were kept at room temperature for 4–6 min for partial evaporation. The slides were then tilted at 20–40 degrees incline in the box for the drop to fall at a constant speed. The box was covered. After 10–15 min of DNA fixation with fresh cold methanol: acetic acid (3:1 or 135 mL/45 mL or 270 mL/90 mL) in the hood, the slides were allowed to dry and stored at 4 °C. On day three of staining, cells were washed twice with 1X PBS. The DNA was denatured for 70 min with 2.5 M Hydrochloric acid at room temperature. The slides were placed in a slow shaker for five minutes; slides were washed three times with 1X PBS for five minutes each. The excess PBS was dapped on paper towels. The slides were blocked with 5% bovine serum albumin/PBS at 37 °C for 30 min. The slides were dipped in 1X PBS for one minute, which was followed by gently sliding the coverslip off. A total of 30 μL of primary antibody cocktail Mouse Anti-BrdU (1/20) and Rat anti-BrdU (1/100) was added in even dots on the slide (in 5% bovine serum albumin/PBS-Tween 20). Coverslips were placed on the slide and incubated in a humid chamber at room temperature for 90–120 min. The slides were then immersed in 1X PBS for 1–2 min to slide the coverslip gently. This was followed by washing 3 times with 1X PBS-Tween 20 in a slow shaker then put in 1X PBS for five minutes each. This was followed by adding 30 μL of secondary antibody. Secondary antibody cocktail (Goat Anti-Mouse Alexa Fluor 488 green (Cat# A-11029; Invitrogen, Carlsbad, NC, USA) at 1/100 dilution and Donkey Anti-Rat Alexa Fluor^®^ 594 red (Abcam, Waltham, MA, USA, Cat# ab150156) at 1:100 dilution. Slides were incubated in a dark humid chamber at room temperature for 60 min. The slides were washed once in 1X PBS, followed by drying of the slides. 4′,6-Diamidino-2-phenylindole dihydrochloride (DAPI) was added to stain DNA, and then coverslips were placed and air dried at room temperature and then stored at 4 °C for analysis.

### 2.11. Flow Cytometry

C4-2B parental and *HOXB13K13A* mutant were stained with γH2AX-A488 (Biolegend, Clone 2F3; San Diego, CA, USA) antibody or γH2AX-APC conjugated antibody (Biolegend; Clone 2F3; Cat# 613415). After staining, cells were fixed with 1% *v*/*v* paraformaldehyde and permeabilized with 0.1% Triton-X-100 for 5 min and blocked with 1X PBS buffer containing 3% *w*/*v* protease-free BSA (incubation buffer). The cells were rinsed twice with the incubation buffer, followed by centrifugation and resuspended in 0.5 mL of 1X PBS, and acquired on LSR Canto and analyzed by the FlowJo software v10.10.

### 2.12. Cell Cycle Analysis

C4-2B or 22Rv1 parental and isogenic *HOXB13K13A* mutants were treated with Etoposide and collected at various time points. Cells were processed for cell cycle analysis by staining with propidium iodide. Cells were acquired on LSR Canto and analyzed by the FlowJo software v10.10.

### 2.13. Statistical Analysis

Statistical analyses were performed using Graph PAD PRISM 9.0 software. Unpaired Student’s *t*-test was used to compare two groups, while analysis of variance (ANOVA) was used for three or more group comparisons. *p* values < 0.05 were considered statistically significant.

## 3. Results

### 3.1. Acetylation of HOXB13 Is Induced by DNA Damage and Persists despite PARP Inhibition

**Figure 1:**
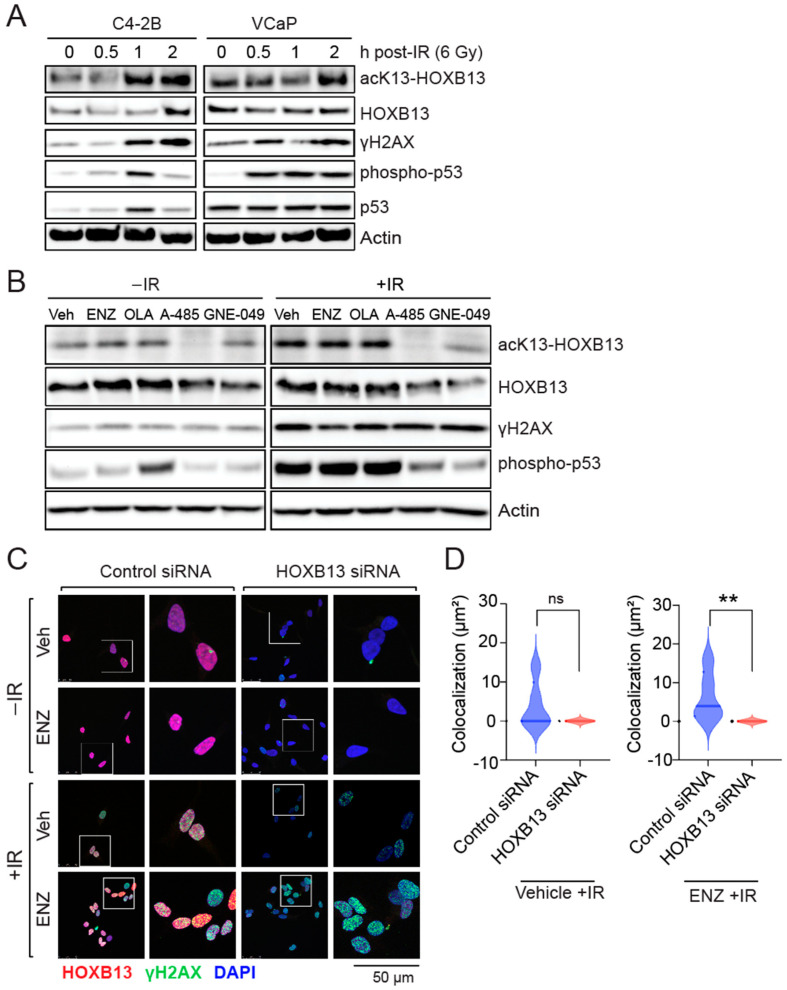
Acetylation of HOXB13 is induced by DNA damage and persists despite treatment with AR antagonist Enzalutamide (ENZ). (**A**) C4-2B and VCaP cells were irradiated (IR) (6 Gy) and harvested at indicated time points. Immunoblot analysis of acK13-HOXB13, pan-HOXB13. γH2AX and p53-pS15 are positive controls for IR treatment. The full pictures of the western blots are included in Supplementary Material. (**B**) VCaP cells were treated with vehicle, ENZ (10 µM), Olaparib (OLA) (2 µM), A-485 (2 µM) and GNE-049 (2 µM) for 24 h. Cells were then treated with IR (6 Gy) and harvested after 2 h. Immunoblot analysis with acK13-HOXB13 and pan-HOXB13 antibodies. H2AX-pSerine139 and p53-pSerine15 are positive controls for IR treatment, while actin is normalization control. (**C**) C4-2B were either transfected with control or HOXB13 siRNA. Cells were either treated with Vehicle (Veh) or 10 μM ENZ for 48 h in the absence or the presence of 6 Gy IR. Immunofluorescence was performed for γH2AX (green foci) and HOXB13 (red foci). DAPI (blue) is a nuclear stain. Colocalized foci are seen as yellow on the overlay. The scale bar is 50 μm. (**D**) The sum of the area of the colocalized foci is shown in Vehicle or ENZ treated with IR. No DDR-induced foci were observed in the absence of IR. ns: not significant ** *p* < 0.008.

In response to DNA DSBs-activated ATM kinase phosphorylates, the histone H2AX at serine 139 (γH2AX) initiates an intracellular signaling cascade [29]. γH2AX acts as a beacon for the recruitment of the Mre11/Rad50/Nbs1 (MRN) DNA repair factors at DSB sites. ATM kinase also phosphorylates the tumor suppressor p53 at serine 15 to cause cell cycle arrest. To understand the role of gain of function HOXB13 acetylation in response to DNA damage in PC, we first performed kinetics of HOXB13 acetylation following irradiation (IR)-induced DNA damage in androgen-independent C4-2B and androgen-dependent VCaP cell lines (Figure 1). We observed that HOXB13-K13 acetylation is increased in a time-dependent manner following DNA damage in both cell lines (Figure 1A). Moreover, this increase correlates with DNA damage induced H2AX serine 139 and p53 serine 15 phosphorylations (Figure 1A).

To determine whether the DNA damage-induced HOXB13 acetylation is also regulated by CBP/p300 histone acetyl transferase [17], we treated VCaP cells with the CBP/300 catalytic inhibitor (A-485) or the CBP/p300 bromodomain inhibitor (GNE-049), followed by IR treatment (Figure 1B). As controls, we treated cells with Enzalutamide and the PARP inhibitor, Olaparib, followed by IR. While the AR antagonist, ENZ or the PARP inhibitor Olaparib did not inhibit DSB-induced HOXB13 acetylation, the CBP/p300 catalytic inhibitor A-485 suppressed it, before and after DNA damage, while the bromodomain inhibitor GNE-049 was more effective after the induction of DSBs (Figure 1B). Additionally, p300/CBP inhibition decreased p53 serine phosphorylation, suggesting a role for p300/CBP in DNA damage-induced checkpoint signaling.

To examine how HOXB13 responds to DNA damage, we examined the distribution of HOXB13 protein in cells undergoing DNA damage. We observed that HOXB13 colocalizes with γH2AX at sites of DSBs following DNA damage by IR and this colocalization persists in cells despite suppression of AR transcriptional activity with Enzalutamide (Figure 1C,D; Supplementary Figure S1), suggesting an androgen-independent role of HOXB13 in the DNA damage response.

### 3.2. HOXB13 Acetylation Promotes Radioresistance of PC Cells

**Figure 2:**
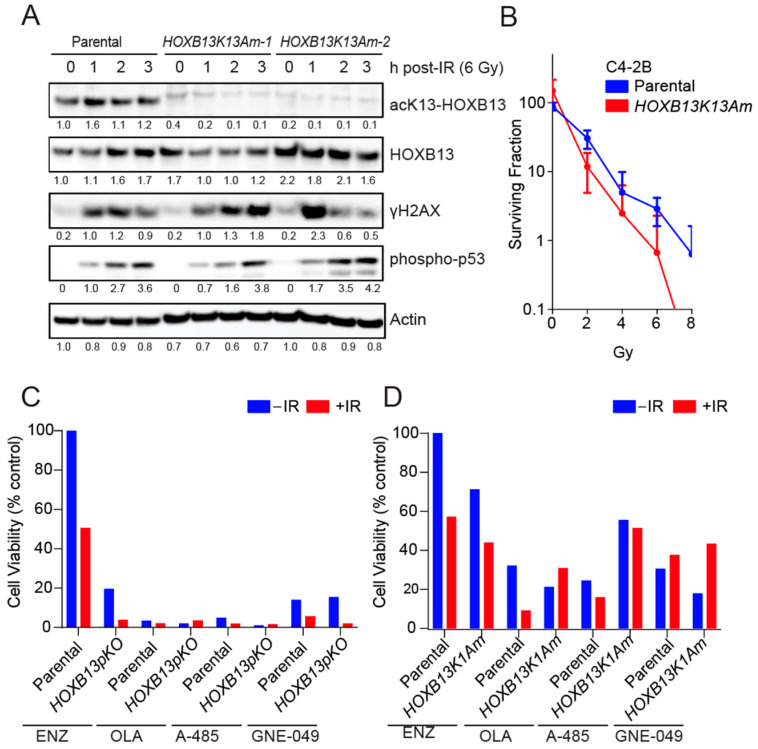
*HOXB13* deletion or loss of acetylation radiosensitizes ENZ-resistant PCs. (**A**) C4-2B-parental and *HOXB13K13Am* cells were irradiated (IR) and harvested at the indicated time points. Immunoblot analysis of acK13-HOXB13, pan-HOXB13. H2AX-pS139 (γH2AX) and p53-pS15. Actin is normalization control. The full pictures of the western blots are included in Supplementary material. (**B**) Clonogenic survival assay: C4-2B-parental and *HOXB13K13Am* cells were either untreated or treated with increasing doses of radiation and plated for single colonies in a 6-well plate (*n* = 6 replicates). Colonies formed were counted after ~12 days and the surviving fraction was calculated. (**C**,**D**) Cell viability assay following treatment with ENZ, Olaparib (OLA), A-485 and GNE-049 inhibitors to compare the sensitivity of parental and *HOXB13pKO* or *HOXB13K13Am* mutants, with and without ionizing radiation (IR) treatment. After a total of 96 h of inhibitor treatment, cell viability was determined using CellTiter-Glo (Promega) (*n* = 8 replicates/condition). IC_50_ results are normalized to ENZ-treated parental control (Also see Supplementary Figures S2 and S3).

Clinically, ADT-treated mCRPC patients with Homologous DNA Repair (HDR) defects respond better to PARP inhibition (88% in HDR deficient, versus 33% in HR proficient) with longer progression-free and overall survival [30]. To further investigate the role of HOXB13 acetylation in DNA damage response, we performed immunoblot analysis of the isogenic parental and *HOXB13K13A* mutant cell lines. This analysis revealed that HOXB13-K13 acetylation was undetectable in *HOXB13K13A* mutants before and after IR (Figure 2A). However, the peak H2AX-pS139 levels differed in the *HOXB13K13A* mutants compared to the parental controls. Moreover, the total HOXB13 levels increased in response to parental DNA damage, but decreased in *HOXB13K13A* mutant, suggesting that HOXB13-K13 acetylation maintains HOXB13 levels in cells responding to DNA damage.

Clonogenic survival assays revealed *HOXB13K13A* mutants were significantly radiosensitive compared to their parental controls (Figure 2B; Supplementary Figure S2A). To further examine the role of HOXB13 in conferring radioresistance, we treated the mCRPC line C4-2B with increasing concentrations of anti-androgen ENZ plus or minus IR and determined cell viability. As expected, we observed that C4-2B are highly resistant to ENZ, and this resistance can be overcome partly by treatment with IR (100 μM vs. 40.5 μM) (Figure 2C; Supplementary Figure S3A). Complete genetic ablation of *HOXB13* is lethal [10]. Therefore, we used a partial *HOXB13* knockout cell line (*HOXB13pKO*). Reduction in HOXB13 protein levels significantly sensitizes C4-2B to ENZ (100 μM vs. 15.68 μM), which is further synergized by IR (3.10 μM) (Figure 2C; Supplementary Figure S3A; compare right and left panels). In contrast to ENZ, C4-2B are highly sensitive to the PARP1 inhibitor, (Olaparib, 2.7 μM) even in the absence of DNA damage, likely due to p53-induced cell cycle arrest. The sensitivity of parental cells to the PARP1 inhibitor increased slightly after DNA damage (1.6 μM) (Supplementary Figure S3B; compare right and left panels). However, genetic ablation of *HOXB13* did not significantly increase this sensitivity further when tested alone (2.7 μM vs. 1.6 μM) or combined with IR (Supplementary Figure S3B; right panel). In contrast, C4-2B are significantly sensitive to the histone acetyl transferase CBP/p300 inhibitor A-485, and this can be further sensitized by *HOXB13* partial ablation (3.9 μM vs. 0.82 μM) (Supplementary Figure S3C). Reduction of HOXB13 acetylation by pre-treatment with A-485 followed by IR showed a modest increase in sensitivity (3.9 μM to 1.49 μM). However, pre-treatment with CBP/p300 bromodomain inhibitor GNE-049 was not as effective as the catalytic inhibitor A-485 in the absence of IR but did increase the sensitivity of *HOXB13pKO* after IR (Supplementary Figure S3D). To specifically confirm that it is the HOXB13 lysine 13 acetylation regulating sensitivity to IR, we tested the *HOXB13K13A* mutant (Figure 2D; Supplementary Figure S4). We observed that the *HOXB13K13A* mutant is highly sensitive to a combination of IR and Enzalutamide. (Figure 2D; Supplementary Figure S4A). In contrast, the *HOXB13K13A* mutant is resistant to Olaparib compared to parental control, suggesting that sensitivity to PARP inhibition may be dependent on HOXB13 acetylation functions in PC cells (Figure 2D; Supplementary Figure S4B). Both *HOXB13K13A* mutants and parental cells displayed similar levels of sensitivity to the CBP/p300 inhibitor A-485 or GNE-049 (Figure 2D; Supplementary Figure S4C,D), suggesting that in prostate cancer, HOXB13 lysine 13 acetylation confers a therapeutic vulnerability to DNA-damaging therapies.

### 3.3. HOXB13-K13 Acetylation-Defective Mutants Show Increased DNA Damage and Impaired Replication Fork Progression

**Figure 3:**
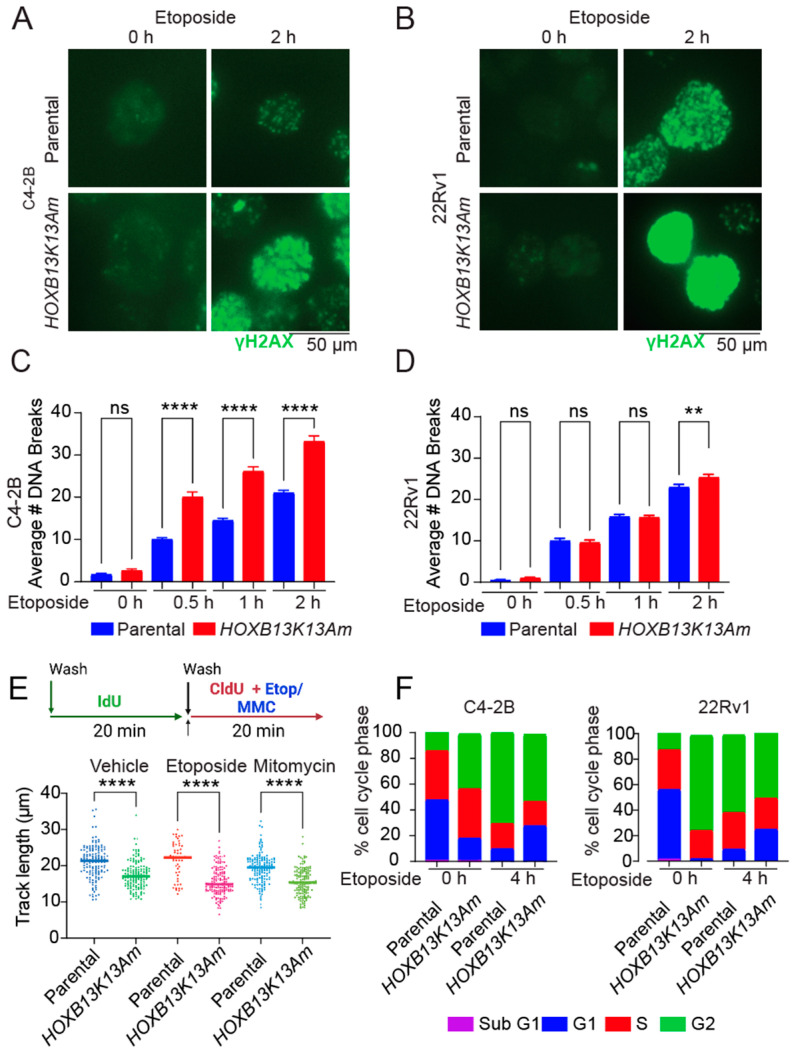
*HOXB13K13A* mutants show an increase in DNA damage following treatment with Etoposide. (**A**) Parental C4-2B and isogenic *HOXB13K13A* mutant or (**B**) Parental and isogenic *HOXB13K13A* mutant 22Rv1 were treated with 10 μM Etoposide followed by staining for γH2AX foci at 0 and 2 h. (**C**,**D**) Quantitation of DSBs for C4-2B and 22Rv1 is shown; ANOVA ** *p* < 0.01, **** *p* < 0.005, ns: not significant. (**E**) Replication fork progression is inhibited in the *HOXB13K13A* mutants in response to DNA damage induced by Etoposide (200 nM) or mitomycin C (MMC; 2.5 μM); ANOVA **** *p* < 0.005. The schematic shows the sequential incorporation of IdU (20 min) and CldU plus treatments (20 min). (**F**) C4-2B and 22Rv1 were treated with 10 μM Etoposide and harvested after 4 h for cell cycle analysis. Cells in different phases of the cell cycle are shown.

Consistently, we observed high levels of γH2AX -signals in response to DNA damage induced by the topoisomerase inhibitor Etoposide in the *HOXB13K13A* mutant at 2 h post-treatment compared to the isogenic C4-2B and 22Rv1 parental cell lines (Figure 3A,B). Immunoblot showed delayed γH2AX formation in the mutant following etoposide treatment (Supplementary Figure S5). Quantitative analysis revealed an increased accumulation of γH2AX-positive DSBs in the mutants compared to the parental controls by two hours (Figure 3C,D). To determine the effect of these accumulated DSBs on DNA replication in the *HOXB13K13A* mutants, we examined tract length at the replication forks using DNA fiber analysis [31,32]. We analyzed track lengths following the incorporation of IdU for 20 min followed by CldU plus Etoposide or mitomycin C (MMC). DNA fiber analysis revealed that *HOXB13K13A* mutants had shorter DNA track lengths compared to their parental counterparts, and this was exacerbated in these cells following treatment with Etoposide and mitomycin C (Figure 3E). Moreover, in contrast to parental cells, the *HOXB13K13A* mutants showed reduced G2/M arrest (Figure 3F). Overall, these results indicate a direct role for HOXB13 in facilitating cell cycle progression in response to DNA damage.

### 3.4. N-Terminal Disordered Region of HOXB13 Forms Nuclear Puncta and Confers Resistance to DNA Damage

**Figure 4:**
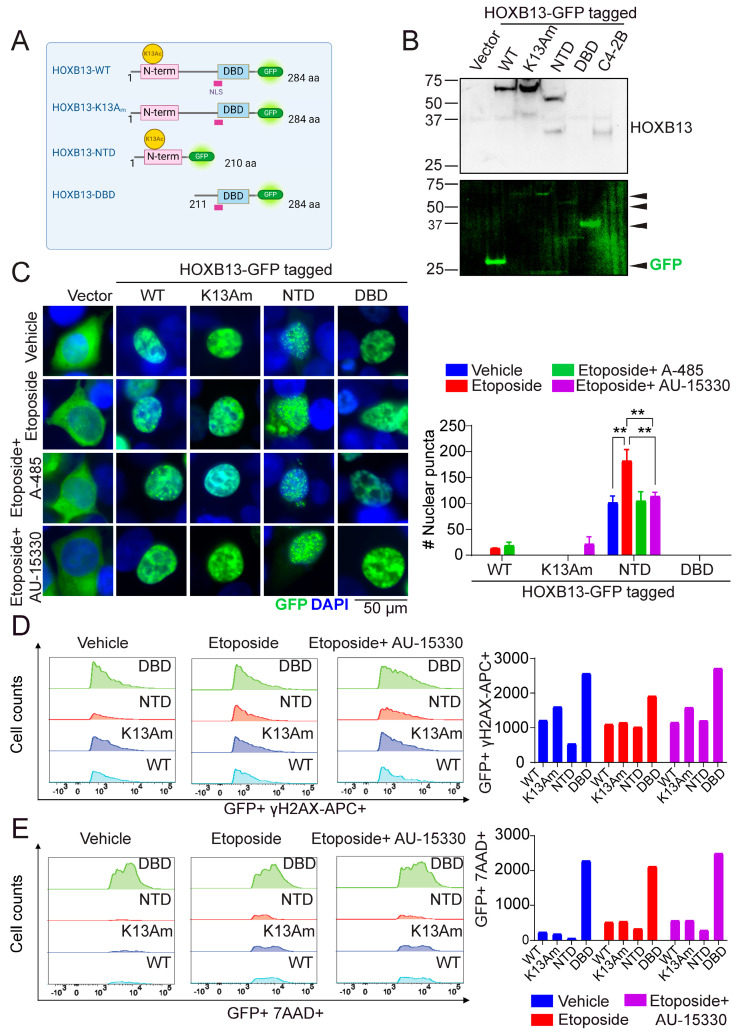
HOXB13 N terminal region forms nuclear puncta that is enhanced by DSBs. (**A**) Schematic of HOXB13 WT and mutant-GFP tagged constructs. NLS: Nuclear localization signal (214-RRGRKKRIPYS-223). DBD/HD: Homeobox domain. (**B**) HEK293T cells transfected with Green Fluorescent Protein (GFP)-Vector, HOXB13-WT-GFP, HOXB13-K13A-GFP, HOXB13-NTD (N-terminal domain) GFP or the HOXB13-DBD (DNA binding) (217–277)-GFP mutant. Arrows indicate GFP and GFP-tagged HOXB13 proteins. Top panel: Blotting with HOXB13 monoclonal antibody (does not recognize DBD); C4-2B-PC cell line with HOXB13 expression. Bottom panel: Green fluorescence to show fusion constructs. (**C**) Visualization of the GFP-tagged proteins in HEK293T cells treated with Vehicle (DMSO) or Etoposide (10 μM) or Etoposide (10 μM) + A-485 (2 μM) or Etoposide + AU-15330 (100 nM) by fluorescence microscopy. The nuclei were counterstained with DAPI shown in blue (left). Scale bar 50 μm. Quantitation of the nuclear puncta for each condition and different treatments (right). ANOVA ** *p* < 0.01. (**D**) Flow cytometry analysis of GFP-positive and γH2AX-APC double-positive cells to measure levels of DSBs. (**E**) Flow cytometry analysis of GFP-positive and 7-AAD-perCP-Cy5.5 double-positive cells to measure viability.

HOXB13 lysine 13 acetylation site is unique to HOXB13 and is not in the other human HOX13 paralogs indicating some non-redundancy in function of this acetylation. To understand how each region is critical for HOXB13 function in PC, we generated deletion constructs of HOXB13 that express either only the N-terminal domain with acetylation site (NTD) or only the DNA binding domain (DBD) with full-length WT-HOXB13 and K13-mutant HOXB13 as controls (Figure 4A). Expression of the constructs is shown in (Figure 4B). A predicted nuclear localization signal (NLS:214-RRGRKKRIPYS-223) is located at the DNA-binding homeobox domain of HOXB13. NLS with basic amino acid clusters are predicted to be present in the homeodomain of a large number of the *HOX* family members [33]. We observed that in response to DNA damage induced by Etoposide, WT-HOXB13 forms nuclear foci as previously noted with irradiation (Figure 4C). Interestingly, the NTD that has an intrinsically disordered region with no known NLS is not only able to translocate to the nucleus but also forms distinct nuclear puncta with an increase in numbers following DNA damage (Figure 4C). Treatment with the CBP/p300 HAT inhibitor A-485 or the SWI/SNF inhibitor AU-15330 reduced the DNA-damage-induced HOXB13 NTD nuclear puncta (Figure 4D). This puncta formation appears to be a unique feature of the NTD as the DBD of HOXB13 does not show puncta formation in the absence or presence of DNA damage despite its nuclear localization (Figure 4B–D).

**Figure 5:**
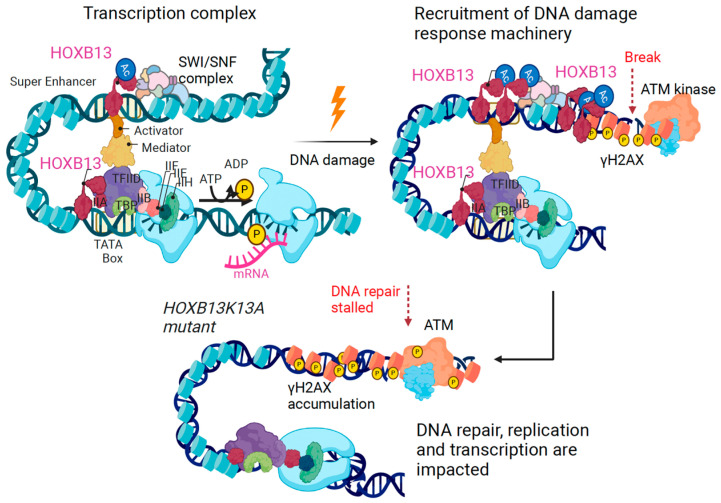
Model: HOXB13 acetylation is critical for DNA damage response. In *HOXB13K13A* mutants, the accumulation of unrepaired DNA breaks eventually lead to cell death.

Next, we examined how cells expressing NTD and DBD respond to DNA damage by measuring γH2AX levels and viability through the incorporation of DNA binding compound 7-AAD (7-amino-actinomycin D), which is taken up by dead cells but excluded by intact viable cells during flow cytometric analysis. Cells expressing the GFP tagged full-length HOXB13, or the mutant constructs were treated with Etoposide, alone or with Etoposide plus CBP/p300 inhibitor A-485 or Etoposide plus SWI/SNF inhibitor AU-15330 followed by flow cytometry analysis for co-staining for GFP with γH2AX-APC (Supplementary Figure S6A) or GFP with 7AAD (Supplementary Figure S6B). We observed that NTD is protective of DNA-damage-induced cell death as seen in a decrease in co-staining for γH2AX phosphorylation and reduced 7-AAD positive cells compared to the extensive damage and loss of viability in the DBD mutant (Figure 4E). Our results suggest that HOXB13 K13 acetylation regulates nuclear condensate formation, which may be essential to facilitate repair (Figure 5).

## 4. Discussion

Prostate cancer cells rely on AR, HOXB13 and FOXA1-regulated transcriptional programs for tumor progression and resistance to anti-androgens [18,34]. In situations of genotoxic stress, an efficient DNA damage response (DDR) is critical to ensure timely DNA repair to ensure genomic integrity and avoid mitotic catastrophe prior to cell cycle progression [29]. While mutations in DNA repair and DNA damage checkpoint genes contribute to the onset of various malignancies [35], cancer cells hijack DNA repair pathways to overcome and adapt to genotoxic insults. In addition, tissue-enriched transcription factors and chromatin remodeling proteins can converge and collaborate with the DNA damage response machinery at sites of DNA damage and stalled replication forks to facilitate or inhibit repair [36,37]. We uncovered that K13 acetylated HOXB13 is induced rapidly in cells in response to radiation-induced DNA damage localized with γH2AX at sites of DNA damage. Genetic or pharmacological ablation of HOXB13 acetylation sensitizes cells to radiation, suggesting a critical role for this modification in restoring genomic integrity and promoting survival. The loss of function *HOXB13K13A* mutants cells are not only significantly sensitive to DSB-inducing agents, but also to cross-linking agents such as DNA mitomycin C. Clinically, DNA cross-linking agents such as cisplatin or carboplatin are used for platinum-based treatments, and these have shown anti-tumor activity in a subset of biomarker-positive advanced PC patients with DNA repair gene aberration [5]. Although we did not specifically test cisplatin and carboplatin in our study, based on the mechanisms of action of DNA cross-linking agents, we expect *HOXB13K13A* mutants to be sensitive to them.

This histone acetyl transferases CBP/p300 acetylate HOXB13 following DNA damage. Moreover, DNA damage-independent and damage-dependent HOXB13 acetylation likely occurs in the nucleus due to enrichment of the proteins at genomic loci associated with transcriptional regulation. The lysine 382 in the tumor suppressor p53 is also a target of CBP/p300 acetylation in response to various stresses such as UV-B irradiation or proteasome inhibitors, and this modification appears to contribute to p53 stability [38]. Comparison of the kinetics of HOXB13 expression following DNA damage revealed a buildup of total HOXB13 in the parental cell line compared to the *HOXB13K13A* mutant, suggesting that this acetylation may contribute to HOXB13 stability.

Earlier, we reported that HOXB13 is an epigenetic target of BRD4 in CRPCs, and suppression of HOXB13 expression by the BET inhibitors JQ1 or MA4-class of compounds induces apoptosis and significantly inhibits PC xenograft tumor growth [10,15,39]. Li et al. have shown that BET inhibitor treatment can enhance the efficacy of radiotherapy and overcome the radioresistance of mCRPCs [40]. As HOXB13 is a direct target of BRD4, the synergy of BETi with radiation treatment in CRPCs is not completely unexpected due to the significant decrease in HOXB13 expression, thus phenocopying *HOXB13pKO* radiation sensitivity in our current study. Thus, the impaired replication fork progression and cell death in the HOXB13K13 acetylation defective mutants reveal a novel epigenetic regulatory mechanism regulating the DNA damage response in PC.

While emerging studies have identified the functional significance of chromatin remodeling proteins and transcription factors in resistance to anti-androgens, how these proteins co-coordinate activities to respond to genotoxic stress is less well-defined [41]. The SWI/SNF chromatin remodeling complex is a large and versatile protein complex, composed of many subunits, which regulates replication and transcription, as well as facilitates repair of DSBs by homologous recombinational repair or non-homologous end joining; thus, it has a potential role as a tumor suppressor [34,42,43]. However, gain-of-function in some of the subunits or interacting proteins such as HOXB13 may underlie malignancy due to rewiring of the complex activities. Thus, acetylated HOXB13 at superenhancers of actively transcribed regions may act as a liaison between the SWI/SNF chromatin remodelers and the DDR machinery and benefit cancer cells following DNA damage.

**Figure 6:**
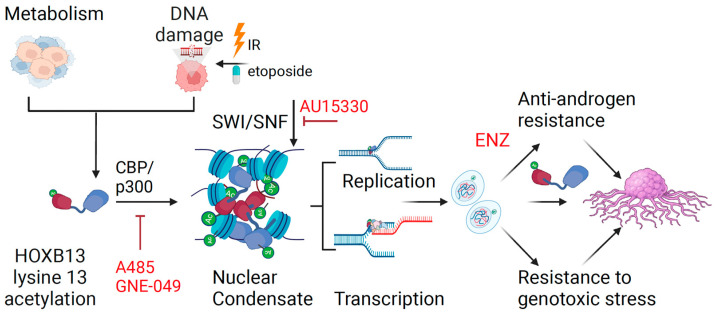
Mechanism of action of HOXB13 acetylation in regulating transcription and DNA damage response in prostate cancer. DNA damage and metabolic alterations impinge on HOXB13 to promote CBP/p300-mediated HOXB13 lysine 13 acetylation. Acetylated HOXB13 interacts with chromatin remodeling enzymes to facilitate DNA repair, replication and transcription. Consequently, HOXB13 promotes cell proliferation and tumor growth defying AR antagonism. Inhibitors of the HOXB13-regulated DDR are shown in red.

Recent studies have demonstrated that disrupting SWI/SNF with selective inhibitors can overcome the resistance of some cancers including CRPCs to targeted therapies [43,44,45,46]. An emerging mechanism gaining traction in the field is the formation of biomolecular condensates formed through liquid–liquid phase separation (LLPS), which promotes intermolecular interactions between transcription factors, coactivators and chromatin remodeling proteins at super enhancers [47,48,49,50]. Increased HOXB13 acetylation is a hallmark of tumor-specific super enhancers and genes associated with increased transcriptional activity and interacts with SWI/SNF chromatin remodeling complex [17]. In our study, we observed that the intrinsically disordered amino terminus of HOXB13 forms distinct nuclear puncta when separated from the ordered DNA-binding homeodomain. Moreover, acetylation of HOXB13 at lysine 13 regulates condensate formation in response to DNA-damaging agents. Conversely, treatment with SWI/SNF inhibitor reduced the number and size of the HOXB13 condensates. Interestingly, we observed that the DNA-binding domain inhibits HOXB13 condensate formation in solution. However, this inhibition can be overcome by full-length HOXB13 following exposure to genotoxic stress. The stages of DNA damage response where HOXB13 activity can be pharmacologically targeted are illustrated in Figure 6. 

## 5. Conclusions

We demonstrate a novel mechanism by which HOXB13 promotes radio- and chemoresistance of prostate cancers. Acetylated HOXB13 has an essential function in maintaining genome integrity and cell survival through its ability to regulate nuclear condensate formation. Future research could explore drugs that can disrupt HOXB13 condensation and thereby compromise the oncogenic activity of HOXB13.

## Data Availability

Data is contained within the article or Supplementary Materials.

## Supplementary Materials

The following supporting information can be downloaded at: https://www.mdpi.com/article/10.3390/cancers16091622/s1, Figure S1: HOXB13 is recruited to DNA double strand breaks. Figure S2: *HOXB13K13A* mutants show impaired colony forming ability after DNA damage. Figure S3: Sensitivity of *HOXB13pKO* mutants to combination treatments. Figure S4: Sensitivity of *HOXB13K13A* mutants to combination treatments. Figure S5: HOXB13 acetylation levels in response to etoposide treatment of prostate cancer cells. Figure S6: Flow cytometry analysis of HOXB13-GFP positive γH2AX-APC positive cells and HOXB13-GFP positive 7AAD positive cells. The uncropped blots and molecular weight markers are shown in attached Supplementary File. The uncropped blots/Full pictures of the Western blots are shown for Figure 1A,B, Figure 2A and Figure 4B and for Supplementary Figure S4A.
