# Supplementary material for: Amino Terminal Acetylation of HOXB13 Regulates the DNA Damage Response in Prostate Cancer
Source: Cancers (Basel). 2024 Apr 23;16(9):1622. doi: 10.3390/cancers16091622 (PMC11083449; doi:10.3390/cancers16091622)
Supplement: Supplementary file 1 [file cancers-16-01622-s001.zip › cancers-2954029-supplementary.pdf]

Supplementary Figure S1

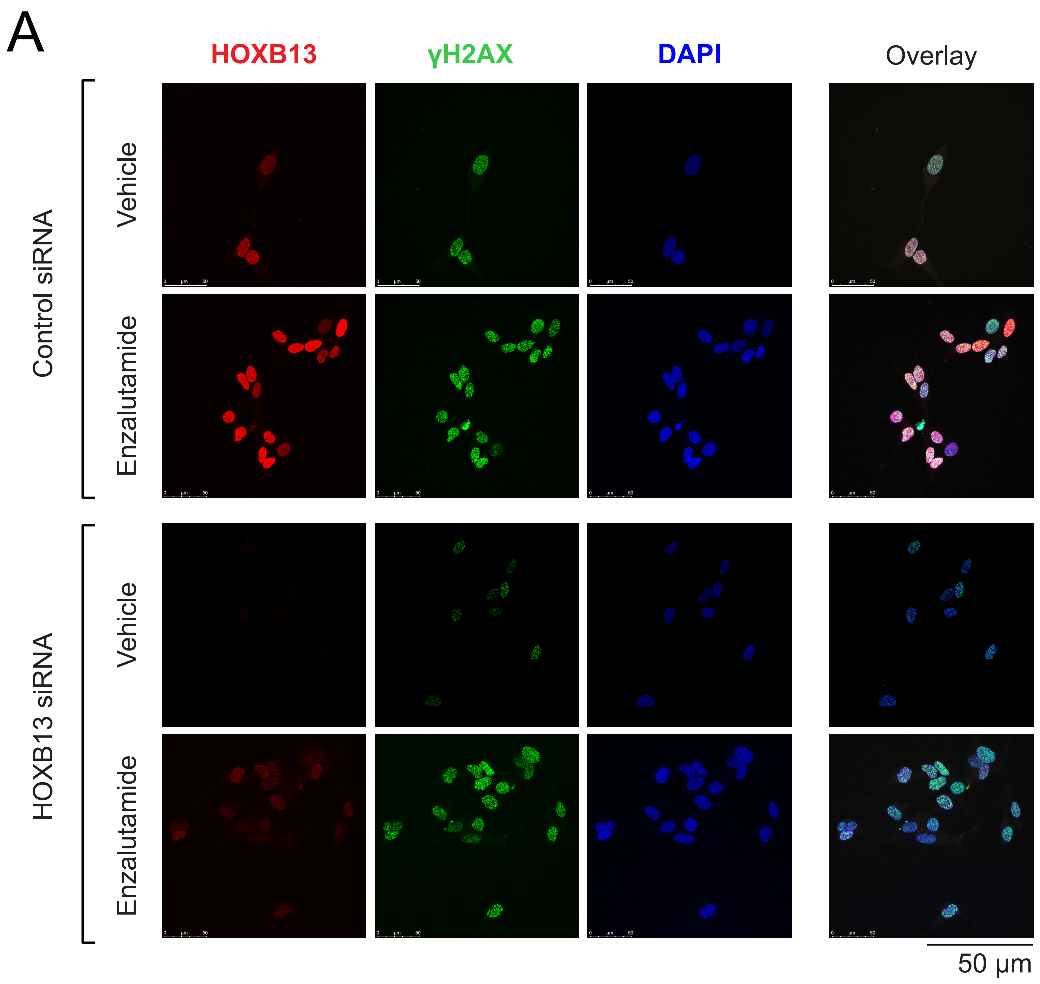

Supplementary Figure S1. **HOXB13 is recruited to DNA double strand breaks.**
C4-2B cells were irradiated at 6 Gy and fixed after 30 mins. Cells were stained for HOXB13 (red), γ-H2AX (green), DNA (blue) and overlay (yellow-orange). DNA damage induced foci were visualized using a confocal microscope. Scale Bar 50 μm.

Supplementary Figure S2

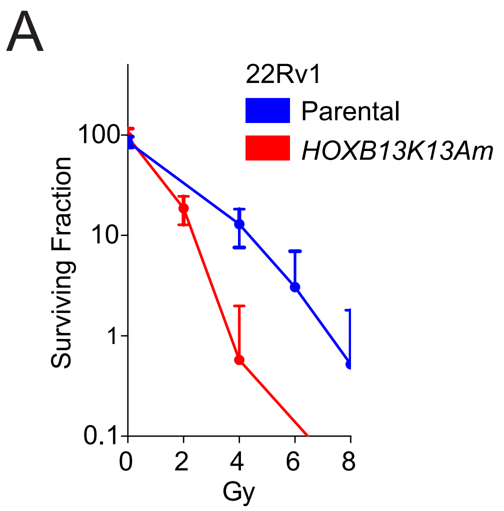

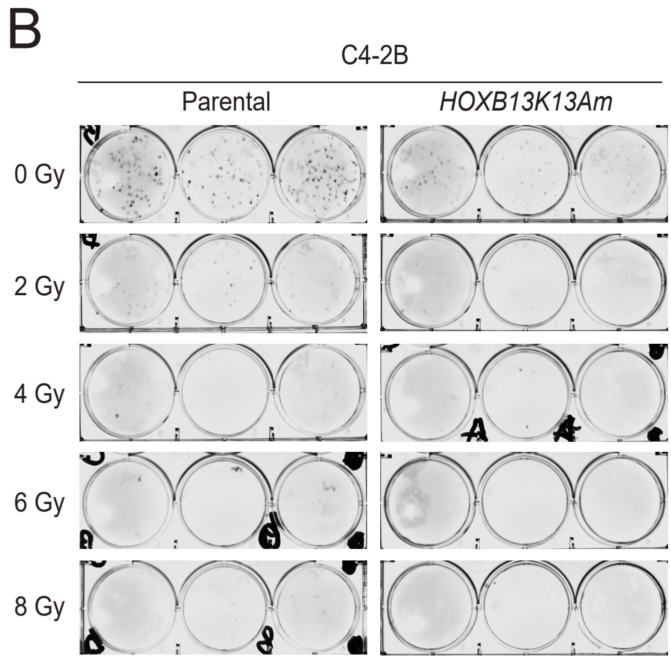

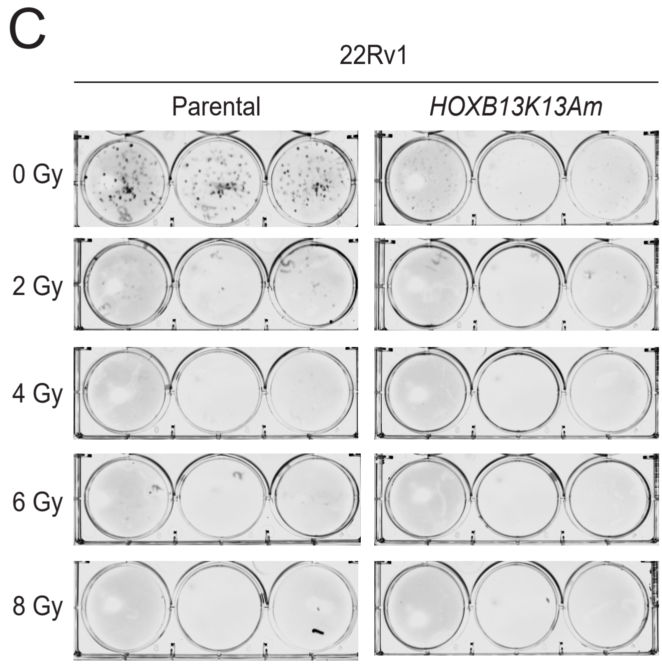

Supplementary Figure S2. ***HOXB13K13A* mutants show impaired colony forming ability after DNA damage.**

(A) 22Rv1 parental and *HOXB13K13Am* cells were given increasing doses of radiation (0-8 Gy) and plated in 6 well plates to obtain individual colonies (n=6). Colonies were stained with 0.5% crystal violet after ~12 days and surviving fraction was calculated. **(B-C)** Representative images of colonies formed are shown for parental and *HOXB13K13Am* at various doses.

Supplementary Figure S3

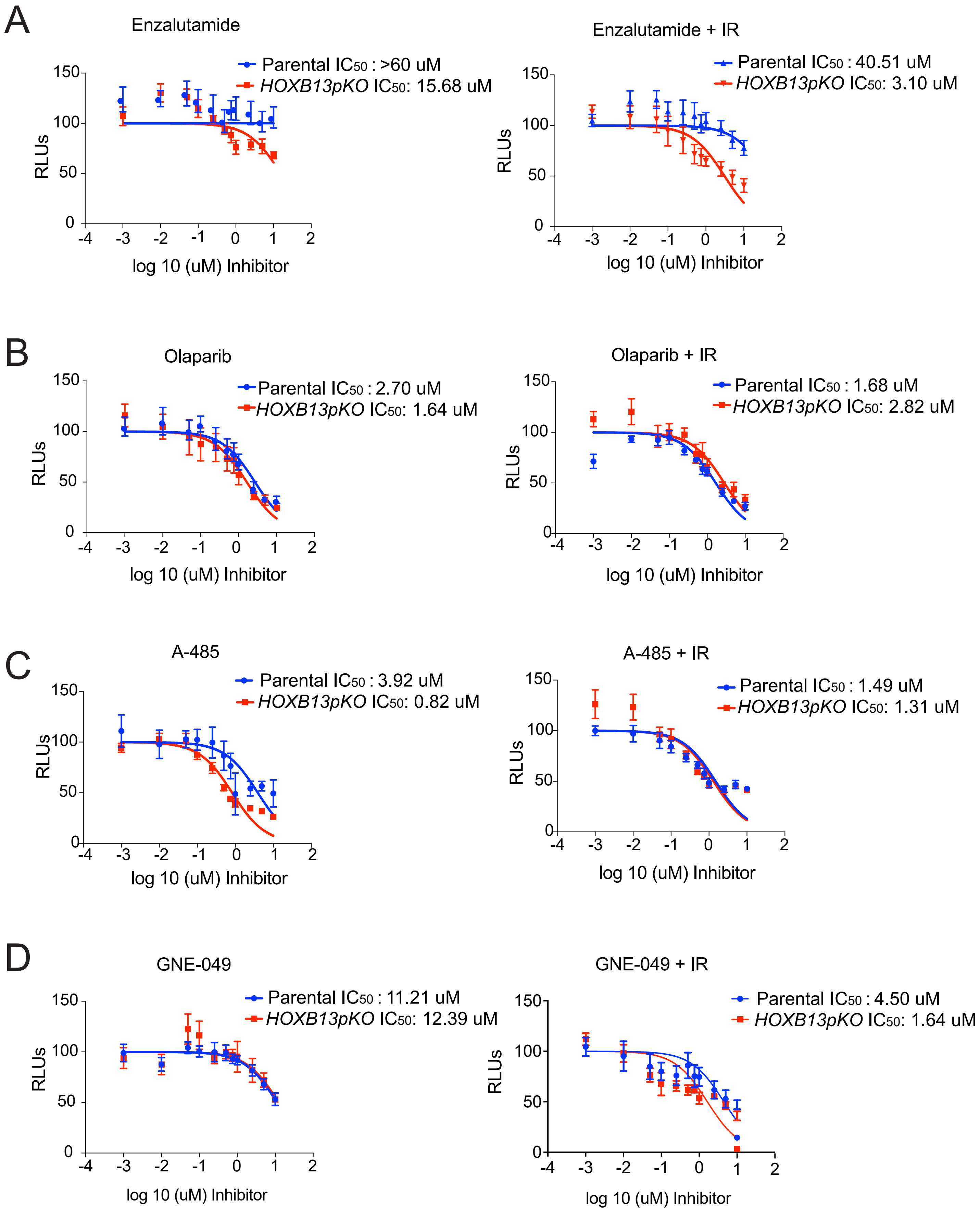

Supplementary Figure S3. **Sensitivity of *HOXB13pKO* mutants to combination treatments.**

Cell viability assay following treatment with Enzalutamide, Olaparib, A-485 and GNE-049 inhibitors to compare the sensitivity of parental and *HOXB13pKO* mutants, minus or plus 6 Gy irradiation treatment.

Supplementary Figure S4

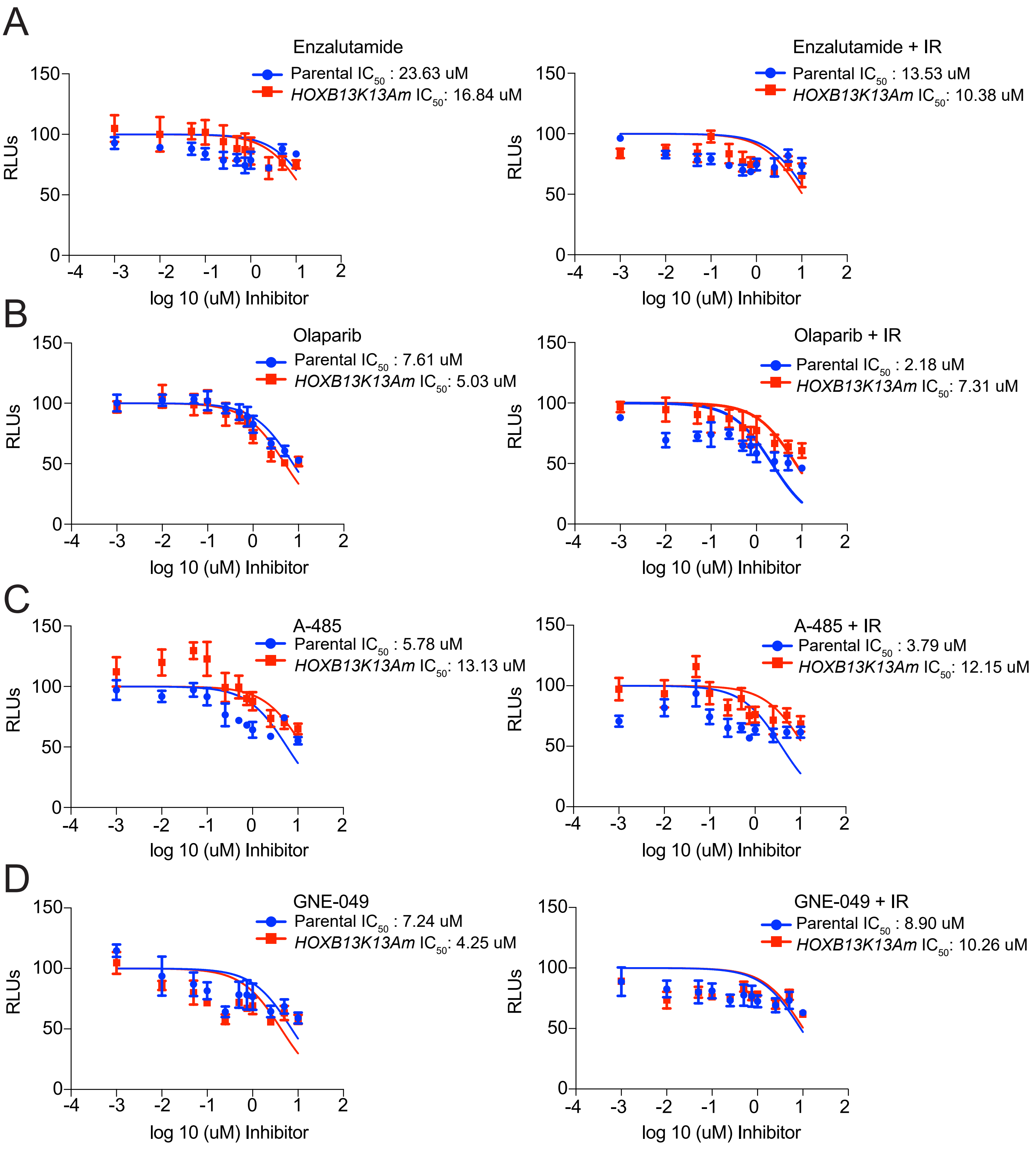

Supplementary Figure S4. **Sensitivity of *HOXB13K13A* mutants to combination treatments.**

Cell viability assay following treatment with Enzalutamide, Olaparib, A-485 and GNE049 inhibitors to compare the sensitivity of parental and *HOXB13K13A* mutants, minus or plus 6 Gy irradiation treatment.

Supplementary Figure S5

A

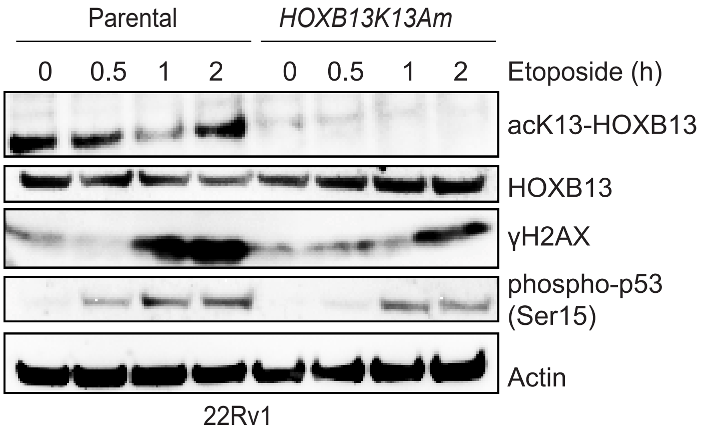

Supplementary Figure S5. HOXB13 acetylation levels in response to etoposide treatment of prostate cancer cells.

Immunoblot analysis with acK13-HOXB13, pan HOXB13, γ-H2AX, p53 serine 15 phosphorylation and actin antibodies in 22Rv1 treated with etoposide. Actin is normalization control.

Supplementary Figure S6

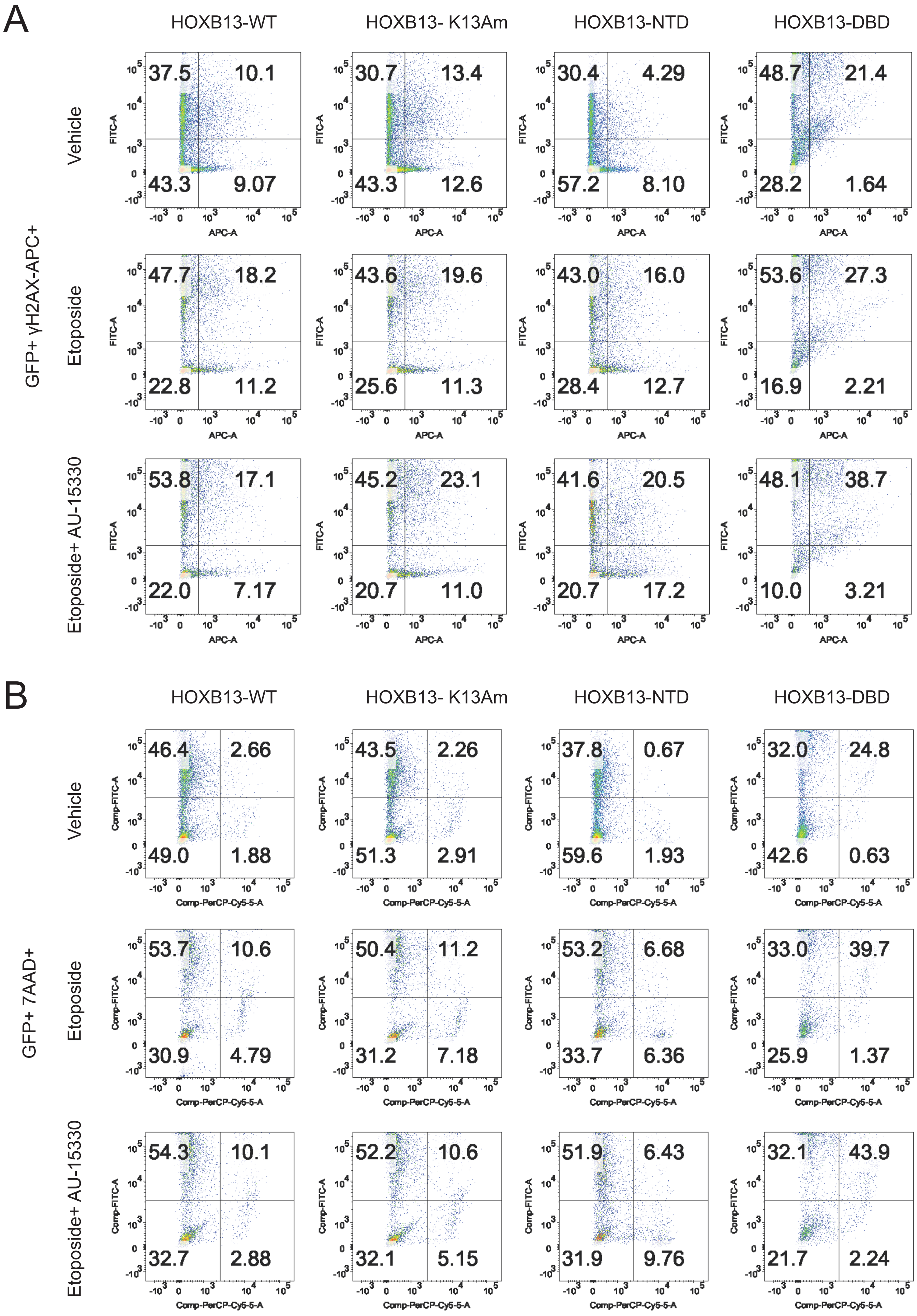

Supplementary Figure S6. Flow cytometry analysis of HOXB13-GFP positive H2AX-APC positive cells and HOXB13-GFP positive 7AAD positive cells.

(A) HEK293T cells transfected with Green Fluorescent Protein (GFP) tagged HOXB13-WT, HOXB13-K13A, HOXB13-NTD (N-terminal domain) or the HOXB13-DBD (DNA binding) GFP mutant with single or combination therapy with etoposide. Cells analyzed for HOXB13-GFP positive and γ-H2AX-APC positive cells. Frequencies of staining are shown for each quadrant.

(B) HEK293T cells transfected with Green Fluorescent Protein (GFP) tagged HOXB13-WT, HOXB13-K13A, HOXB13-NTD (N-terminal domain) or the HOXB13-DBD (DNA binding) GFP mutant with single or combination therapy with etoposide. Cells analyzed for HOXB13-GFP positive and 7-AAD positive cells. Frequencies of staining are shown for each quadrant.
